# Nontuberculous mycobacterial pulmonary disease (NTM PD) incidence trends in the United States, 2010–2019

**DOI:** 10.1186/s12879-024-09965-y

**Published:** 2024-10-02

**Authors:** Samantha J. Bents, Rachel A. Mercaldo, Collin Powell, Emily Henkle, Theodore K. Marras, D. Rebecca Prevots

**Affiliations:** 1 Epidemiology and Population Studies Section, Laboratory of Clinical Immunology and Microbiology, Division of Intramural Research, National Institute of Allergy and Infectious Disease, National Institutes of Health, Bethesda, MD USA; 2 Division of International Epidemiology and Population Studies, Fogarty International Center, National Institutes of Health, Bethesda, MD USA; 3 Oregon Health and Science University, Portland, OR USA; 4 Division of Respirology, Department of Medicine, Toronto Western Hospital, University Health Network, Toronto, ON Canada

**Keywords:** Nontuberculous mycobacteria, Pulmonary disease, Environmental bacteria

## Abstract

**Background:**

Nontuberculous mycobacteria (NTM) are ubiquitous environmental bacteria that cause chronic lung disease. Rates of NTM pulmonary disease (NTM PD) have increased over the last several decades, yet national estimates in the United States (US) have not been assessed since 2015.

**Methods:**

We used a nationally representative population of Medicare beneficiaries aged ≥ 65 years to assess rates of NTM PD in a high-risk population from 2010 to 2019. Poisson generalized linear models were used to assess the annual percent change in incidence in the overall population and among key demographic groups such as sex, geography, and race/ethnicity. We evaluated the relative prevalence of various comorbid conditions previously found to be associated with NTM PD.

**Results:**

We identified 59,724 cases of incident NTM PD from 2010 to 2019 from an annual mean population of 29,687,097 beneficiaries, with an average annual incidence of 20.1 per 100,000 population. NTM PD incidence was overall highest in the South and among women, Asian individuals, and persons aged ≥ 80 years relative to other studied demographic groups. The annual percent change in NTM PD incidence was highest in the Northeast, at 6.5%, and Midwest, at 5.9%, and among women, at 6.5%. Several comorbid conditions were highly associated with concurrent NTM diagnosis, including allergic bronchopulmonary aspergillosis, bronchiectasis, and cystic fibrosis.

**Conclusions:**

Here we provide current estimates of NTM PD incidence and prevalence and describe increasing trends in the US from 2010 to 2019. Our study suggests a need for improved healthcare planning to handle an increased future caseload, as well as improved diagnostics and therapeutics to better detect and treat NTM PD in populations aged ≥ 65 years.

**Supplementary Information:**

The online version contains supplementary material available at 10.1186/s12879-024-09965-y.

## Introduction

Nontuberculous mycobacteria (NTM) are ubiquitous environmental bacteria that cause chronic lung disease [1]. These mycobacteria proliferate in both natural water and soil reservoirs as well as human-engineered water distribution systems, and infection likely occurs via inhalation or aspiration of soil and water aerosols [1, 2]. Host susceptibility is critical to disease pathogenesis following environmental exposure, and predisposing factors for nontuberculous mycobacterial pulmonary disease (NTM PD) include structural, genetic, and immunologic conditions such as cystic fibrosis (CF), non-CF bronchiectasis, and chronic obstructive pulmonary disease (COPD). Older adults aged ≥ 65 years and women are at increased risk of NTM PD [1].

NTM pulmonary infection and disease rates have been increasing in the United States (US) and globally over the past 20 years. A population-based study of hospitalizations in the US from 1998 to 2005 found a significantly increasing rate of NTM PD-associated hospitalizations [3]. The first population-based study of non-hospitalized NTM PD prevalence, defined using International Classification of Disease (ICD) codes, found an increase of 8% per year from 1997 to 2007 among persons aged *≥* 65 years enrolled in Medicare [4]. Subsequent analyses have continued to describe an increase in NTM PD incidence from 2008 to 2015 using microbiologic-based or ICD-code based case definitions [5–7]. Population-based data from Canada, Europe, and East Asia suggest that NTM PD incidence has also increased globally over the past two decades [1]. However, current estimates of the national NTM PD incidence in the US have not been assessed. Here we leverage a population-based dataset of Medicare beneficiaries to provide updated estimates of spatiotemporal NTM PD trends in the US as well as insight into demographic heterogeneity in disease burden among a high-risk population.

## Methods

### Study population

We used the Medicare Carrier Part B claims (medical insurance) dataset provided by the Centers for Medicare and Medicaid Services (CMS) to identify incident NTM PD cases among the full population of beneficiaries ≥ 65 years of age for the study period 2010 through 2019. We extracted all claims associated with the ICD 9th and 10th revision Clinical Modification (ICD-9 and ICD-10) codes for the period 2008 through 2019. Because of irregularities in medical care during the COVID-19 pandemic, claims from 2020 to 2022 were excluded. Persons aged < 65 years were also excluded as were those enrolled in Medicare Part C (Medicare Advantage) administered through an external Health Maintenance Organization (HMO), as their claims may not have been represented in this dataset. Incident cases were defined as having at least two NTM PD (ICD-9 031.0 or ICD-10 A31.0) claims separated by more than one day, but less than 12 months, and with no claims in the prior 24 months. We also described the distribution of NTM PD claims among beneficiaries and assessed trends using a case definition of a single NTM PD claim after a 24-month claim-free period (Supplementary Fig. 1). However, in our primary analysis, we used a definition of two claims to increase the specificity of the case definition and ensure that our study population better represented persons whose NTM PD presented a continuing burden to the healthcare system.

### Statistical analysis

Incidence was calculated by dividing the number of beneficiaries meeting the case definition each year by the total population of enrolled beneficiaries in each group or subgroup at the midpoint of the year. We analyzed NTM PD incidence patterns across four US regions: Midwest, Northeast, South, and West and explored the distribution of cases across key demographic groups: age, sex, and race/ethnicity. In accordance with CMS guidelines, we excluded groups with fewer than 11 beneficiaries to maintain beneficiary privacy.

To assess the current overall NTM PD burden in the population, we estimated prevalence for 2019, the most recent year in our study period. To estimate prevalence, we assumed that the duration of a case of NTM PD was two years, consistent with the recommended treatment period of 12–18 months [8]. Prevalence was calculated by summing all incident cases from 2017 to 2019. In addition, to describe the current etiologies of NTM PD in this population more fully, we estimated the prevalence of selected comorbid conditions among incident cases and beneficiaries in 2019 for rare and common conditions of interest. Beneficiary claims were scanned for at least a single claim with a relevant code at any point during the study period (Supplementary Table 1).

We estimated the annual percent change (APC) in incidence over the study period for each region, race/ethnicity, and sex group using Poisson generalized linear models, where our outcome was the number of incident cases, and the independent variable was the year of the study. We included a population offset for the log of the total number of Medicare beneficiaries in the population of interest in each year, allowing us to control for changes in Medicare enrollment over the study period.

## Results

**Fig. 1:**
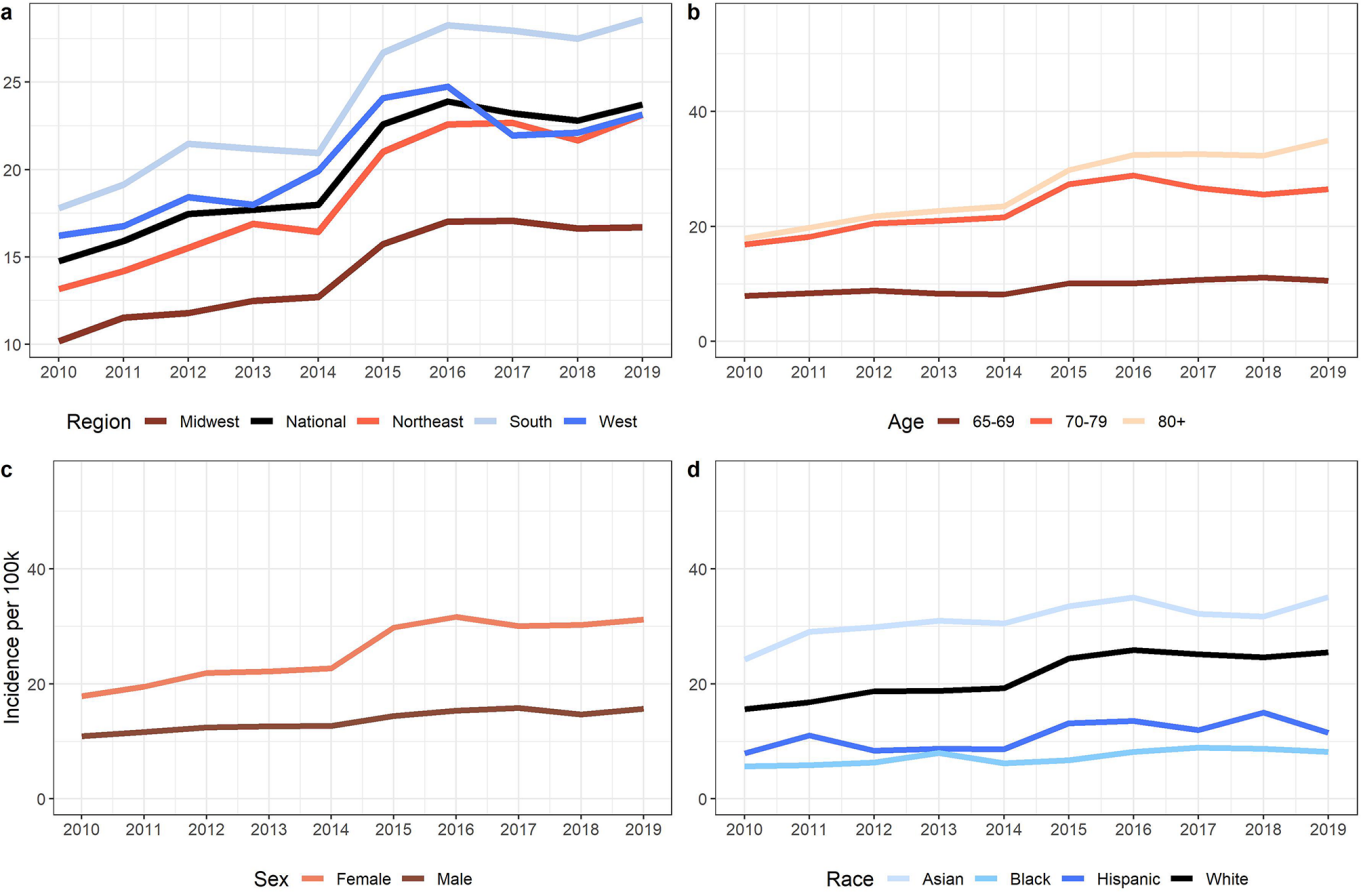
NTM PD incidence trends from 2010–2019. **(a)** Annual incidence time series per 100k Medicare beneficiaries colored by US region (Midwest, Northeast, South, and West) compared to national. Annual national incidence by **(b)** age groups, 65–69-years, 70–79-years, and 80 + years, **(c)** sex, women and men, and **(d)** race, Asian, Black, Hispanic, and White

We identified 59,724 cases of NTM PD from 2010 to 2019 from an annual mean population of 29,687,097 beneficiaries, for an average annual incidence of 20.1 per 100,000 population for the study period. Incidence was highest in the South, rising from 17.8 to 28.6 per 100,000 population from 2010 to 2019 (Fig. 1a). NTM PD trends varied by key demographic factors including age, race/ethnicity, and sex (Fig. 1b-d). We found that NTM PD incidence was increasing significantly across all racial/ethnic groups and among both women and men (Table 1). In 2019, incidence among persons aged ≥ 80 years was 3.3-fold-higher than that for persons aged 65–69 years and 1.3-fold higher than that for persons aged 70–79 years. Women had higher rates of NTM PD than men, with an average annual 1.4- 2.0-fold-higher incidence across age groups during the study period (Supplementary Fig. 2). Among racial/ethnic groups in 2019, incidence among Asian individuals was 1.4-fold higher than that for White individuals, 3.1-fold higher than that for Hispanic individuals. and 4.3-fold higher than that for Black individuals. The APC was highest in the Northeast, at 6.5%, and Midwest, at 5.9%, but increased significantly across all regions.

**Table 1:** Study population and annual percent change (APC) by region, sex, and racial/ethnic group

Population	Average Annual Cases	Midpoint Beneficiary Population (2015)	Annual percent change (95% C.I.)
*Region*			
National	5973	29,399,813	5.5 (5.2, 5.8)
Midwest	973	6,660,629	5.9 (5.2, 6.6)
Northeast	1071	5,717,034	6.5 (5.8, 7.2)
South	2687	11,094,647	5.4 (5.0, 5.9)
West	1243	5,927,503	4.1 (3.5, 4.7)
*Sex*			
Female	4171	16,133,585	6.4 (6.1, 6.8)
Male	1802	13,266,228	4.0 (3.5, 4.5)
*Racial/ethnic groups*			
White	5346	24,856,821	5.7 (5.4, 6.0)
Black	168	2,302,540	4.7 (3.0, 6.4)
Asian	205	641,719	2.7 (1.2, 4.2)
Hispanic	59	516,759	5.3 (2.5, 8.1)

Table 1. **Annual percent change in incidence by geography and demographic factors**. We estimated the annual percent change and 95% confidence intervals by region, sex, and race/ethnicity using Poisson generalized linear models.

**Fig. 2:**
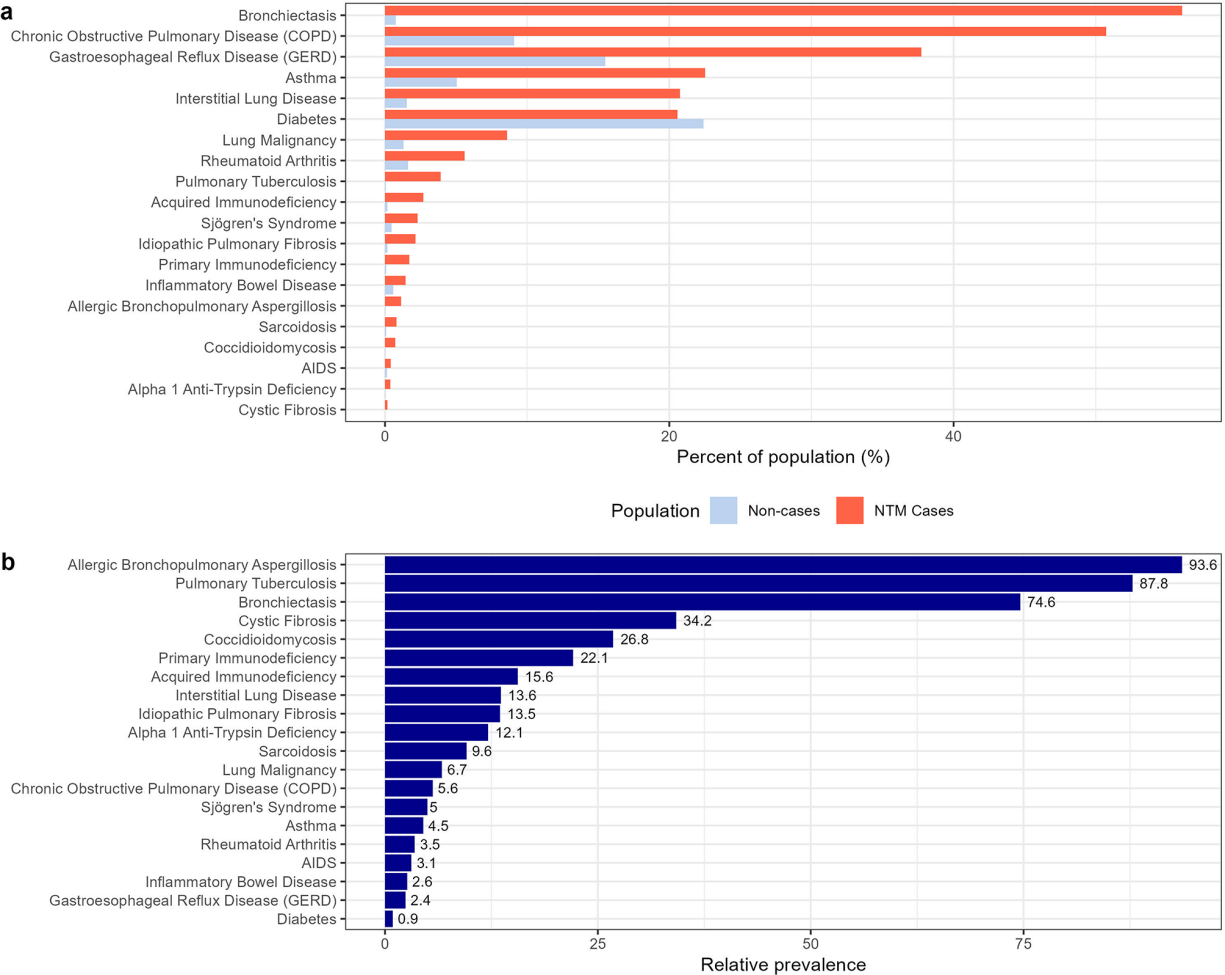
Prevalence of selected comorbid conditions among persons with incident NTM PD in 2019. **(a)** Percent of persons with NTM PD incident cases in 2019 with comorbid conditions compared to the general Medicare population. **(b)** Relative prevalence of comorbid conditions among persons with NTM PD

For 2019, the national prevalence was 91.83 cases per 100,000 population. We further explored the prevalence of selected comorbid conditions among persons with incident NTM PD in 2019 and compared to the prevalence among non-cases in the same year (Fig. 2a). As expected, NTM PD risk was higher among persons with structural, immunological, or genetic conditions affecting lung function, with 94.2% of cases having at least one of the selected comorbidities. However, 437 of 7558 persons (5.8%) with incident NTM PD had no history of any of the selected comorbidities. The absolute prevalence of these selected conditions in cases and non-cases are shown in Fig. 1a, and the relative prevalence comparing cases to non-cases is shown in Fig. 1b. Several conditions were rare overall in the NTM and non-NTM population but had a greatly increased prevalence in the NTM population. For example, although only 1.12% of cases and 0.012% of non-cases had allergic bronchopulmonary aspergillosis (ABPA), the relative prevalence was 93.6, suggested a greatly increased risk of NTM among those with APBA. Similarly, persons with CF have a known increased risk of NTM PD; in this population, persons with NTM PD were 34.2 times more likely to have had at least one claim for CF (Fig. 2b). The relationship between NTM PD and other rare conditions has been less well characterized, but we note here that for several rare comorbid conditions with a prevalence of < 2% in our population, strong associations with NTM PD were observed, including coccidiomycosis (RP = 27), alpha-1 anti-trypsin deficiency (RP = 12), sarcoidosis (RP = 10), lung malignancy (RP = 7), and inflammatory bowel disease (RP = 3). We also observed that associations between NTM PD and other more common conditions known to increase the risk of NTM PD, including COPD (RP = 6) and gastroesophageal reflux disease (GERD) (RP = 2). Of note, the prevalence of diabetes was similar for cases and non-cases (Fig. 2a).

## Discussion

We found that among older adults aged ≥ 65 years in the US, NTM PD incidence has continued to increase. These estimates represent updates on NTM PD trends, with the most recent prevalence estimates being for 2015 [7, 9]. Regional and demographic heterogeneity in disease distribution persists, whereby NTM PD incidence was highest in the South and among women, Asian individuals, and persons aged ≥ 80 years. We also found that NTM PD was increasing significantly nationally, in each region, for both sexes, and all racial/ethnic populations. Our approach, based on ICD codes, represents a conservative estimate, as some proportion of persons with cases in prior years may not respond to treatment or may experience recurrence or reinfection [1]. However, we felt that those instances severe enough to require medical care would be reflected in additional claims.

Our results are consistent with several studies showing nationally and globally increasing rates of NTM PD [1, 10]. In the US, a recent study among persons with CF found a similar annual increase in incidence of 3.5% [11], suggesting that the trends observed for the high-risk CF population are reflective of trends in the older adult population. Several hypotheses may explain these patterns, including increased awareness and detection, rising prevalence of comorbid conditions, and increased environmental risk via climate change.

Rates of some comorbid conditions associated with increased host susceptibility to NTM PD, such as bronchiectasis and asthma, have increased over the same time span, potentially explaining the observed trends [12–14]. However, NTM pulmonary infection and disease have also increased among individuals without underlying conditions, suggesting a role for increased environmental risk that may be driven by climate change [15]. Evidence exists for the influence of severe weather events on NTM infection risk. Several studies have shown an association between hurricanes and flooding events and subsequent NTM infections, potentially linked to increased exposure to stagnant water where NTM proliferates [16, 17]. NTM concentration in the air has also been linked to high speed-wind patterns and dust storms and may be exacerbated by anthropogenic air pollution [18, 19]. Further, ecological analyses have identified associations between rainfall, temperature, and vapor pressure and NTM PD risk [20, 21]. Increased NTM surveillance efforts will allow improved monitoring of NTM infection and disease trends, which may be particularly useful for detecting rises in NTM infection and disease following severe weather events. Further epidemiological studies will be needed to explore the interaction of host susceptibility and the environment in the pathogenesis of NTM PD.

Additionally, rising incidence may also be a product of increased awareness and testing. Prior analyses have found significantly increasing testing rates among different populations; for example, among adults aged *≥* 65 years old attending a diverse group of US health systems, thoracic CT scanning increased by approximately 5% annually from 2013 to 2016 [22], closely mirrored by acid-fast bacilli (AFB) culturing for NTM, which increased at an annual rate of 4.5% in US healthcare settings from 2009 to 2015 [6]. Our study relies on claims in the Medicare beneficiary population, so we could not directly evaluate whether testing rates increased during the study period, but these findings suggest that increased awareness with clinical suspicion of NTM PD may play a role in the trends we have presented.

Our analysis demonstrated that several rare and common conditions were associated with increased NTM PD risk, which may have implications for improved screening among high-risk populations. While it is recommended that persons with CF are tested for NTM at a minimum yearly interval, guidance for bronchiectasis is less prescriptive, suggesting “regular” screening for pathogens that include NTM [8, 23, 24]. No such recommendations exist for any of the other established comorbidities. Our results suggest that additional high-risk populations exist which may benefit from higher frequency testing, including those with rare conditions such as allergic bronchopulmonary aspergillosis, coccidiomycosis, idiopathic pulmonary fibrosis, Sjogren’s Syndrome, interstitial lung disease, and bronchiectasis, as well as common conditions such as COPD, GERD, and asthma. A comprehensive screening approach could include common diseases, even those with modest relative prevalences, which likely translate to many affected patients, and rare diseases with high associated risks, wherein the likelihood of identifying cases is high. An economically driven approach would on the other hand focus on the likelihood of identifying cases by targeting the highest risk conditions. In some instances, these comorbid conditions may share an underlying common etiology with NTM PD. For other more non-specific conditions, such as asthma and interstitial lung disease, some proportion of persons with these conditions may have had a code initially assigned in the NTM PD diagnostic process. Given the long delay and frequent initial misclassification of NTM PD, future work will be needed to elucidate the role of these comorbid conditions more clearly in both the pathogenesis and diagnosis of NTM PD.

Our study has several limitations. First, our analysis relies on claims to identify cases, so without microbiological or radiographic data, we cannot apply the full diagnostic criteria recommended in American Thoracic Society guidelines [8, 23]. However, prior studies have validated claims alongside microbiological testing and found comparable trends. These studies have shown that relative to microbiological criteria, the sensitivity of claims is 27% in the general population and ranges between 50 and 69% in higher-risk populations [5, 25, 26]. Claims are highly specific but less sensitive, and thus using ICD codes tends to underestimate true disease incidence, suggesting that our results are likely conservative [1]. The trends we have presented are also subject to changes in ICD codes, which may explain the observed plateau in incidence observed nationally and across most regions in 2015. ICD codes changed from ICD 9 to 10 in October 2015 [27], which may have resulted in differential diagnostic patterns. Despite the shift in patterns observed in this year, incidence continued to increase through the remainder of the study period, suggesting that our results reflect a true increase over time. However, the continued effect of these changes in ICD codes should be evaluated in future studies. An additional limitation is that because the Medicare racial/ethnic codes are aggregated into broad groups, namely “Asian”, “White”, “Black” and “Hispanic”, we could not examine trends within subgroups, particularly the high risk “Asian” subgroup which comprises multiple subgroups with potentially varying risk. In addition, although sample size constraints prevented us from including age in statistical models, we were able to qualitatively explore age-related effects on incidence patterns.

## Conclusions

Here we provided timely estimates of NTM PD incidence and prevalence and described increasing trends in the US from 2010 to 2019 using a population-based, nationally representative dataset. We explored the geographic and demographic heterogeneity in disease burden and characterized risk among beneficiaries with various high-risk comorbid conditions. Our study, taken together with several prior population-based studies similarly showing increased infection and disease trends, suggests a concurrent increased need for improved healthcare planning to handle an increased future caseload, as well as improved diagnostics and therapeutics to better detect and treat NTM PD. Increased awareness of NTM PD diagnosis and treatment guidelines could lead to improved management of NTM PD.

## Electronic supplementary material

Below is the link to the electronic supplementary material.

Supplementary Material 1

## Data Availability

The data that support the findings of this study are available from the Centers for Medicare and Medicaid Services. Restrictions apply to the availability of these data, which were used under a Data Use Agreement for the current study, and so are not publicly available.
