# Supplementary material for: Nontuberculous mycobacterial pulmonary disease (NTM PD) incidence trends in the United States, 2010–2019
Source: BMC Infect Dis. 2024 Oct 2;24:1094. doi: 10.1186/s12879-024-09965-y (PMC11445848; doi:10.1186/s12879-024-09965-y)
Supplement: Supplementary file 1 — Supplementary Material 1 [file 12879_2024_9965_MOESM1_ESM.docx]

**Supplementary Tables and Figures**

**Supplementary Table 1: ICD9/10 codes for NTM and associated etiologies**

| **Comorbid Conditions** | **ICD-9/10 Codes** |
| --- | --- |
| AIDS | 042, B20, O98.711-O98.713, O98.719, O98.72-O98.73, Z21 |
| Acquired immunodeficiency | D84.821, D84.9 |
| Alpha 1 anti-trypsin | 273.4, E88.01 |
| Allergic bronchopulmonary aspergillosis | 518.6, B44.81 |
| Asthma | 493.90- 493.91-493.92, 493.20-493.22, 493, 493.9, 493.1-493.2, E945.7, 493.11-493.12, 493.00-493.02, 493.82, 493.1,  J45.909, J45.998,  J45.20-J45.22, J45.30-J45.32, J45.40-J45.42, J45.50-J45.52, J45.991,  J45.901-J45.902,  T48.6X5A, J45.90, J45, J45.99, J45.9, T48.6X6A, T48.6X5D, J45.5, J45.4, J45.3 |
| Bronchiectasis | 494, 494.0, 494.1, J47, J47.0-J47.1, J47.9, Q33.4 |
| Chronic obstructive pulmonary disease (COPD) | 496, J41, J42, J44.9, J44.0-J44.1, J44 |
| Cocci | 114.9, 114.0, 114.1, 114.3, 114.5, 114.2, B38.9, B38, B38.0-B38.4, B38.7, B38.81, B38.89 |
| Cystic fibrosis | 277.0, E84, E84.11, E84.19, E84.8-E84.9 |
| Diabetes | 250  E08- E13, E08.00-E08.01 |
| Ehlers-Danlos Syndrome | Q79.6, Q79.60-Q79.63, Q79.69 |
| Gastroesophageal reflux disease (GERD) | 530.11, 530.81, K21, K21.9 |
| Kartagener | 759.3, Q34.8, Q89.3 |
| Idiopathic pulmonary fibrosis | 516.3, J84.112 |
| Inflammatory bowel disease | 555, 555.9, 556.9 K50.90, K51.90, K51.00, K51.919, K51.80, K51.50 |
| Interstitial Lung Disease | 515, 516.0, 516.1, 516.2, 516.30, 516.31, 516.32, 516.33, 516.34, 516.35, 516.36, 516.37, 516.4, 516.5, 516.61, 516.62, 516.63, 516.64, 516.69, 516.9, J84.10, J84.17, J84.170, J84.178, J84.89, J84.01-J84.03, J84.111, J84.112-J84.117, J84.2, J84.81, J84.82, J84.841-J84.842, J84.83, J84.843, J84.848, J84.9 |
| Lung malignancy | 197.0, 162.9, 162.2-162.5, 235.7, 162.8, 162.0, 162, V10.11-V10.12, C34.90, Z85.118, C34.31, C34.10-C34.11, C34.92, C34.91, C33, C34.00-C34.02, C34.32, C34.12,  C78.00-C78.02, C34.2, C34.80, C34.82, D38.1, C34.81, C34.30, Z85.11-Z85.12, C34.1, C34.9,  C34.8, C34 |
| Marfan Syndrome | Q87.4, Q87.40, Q87.410, Q87.418, Q87.42-Q87.43 |
| Primary ciliary dyskinesia | 748.8, 759.3, Q89.3, Q34.8 |
| Primary immunodeficiency | 279.00-279.09, D80.9, D81, D81.1, D81.9, D82.0, D83.0-D83.2, D83.8-D83.9 |
| Pulmonary tuberculosis | 011, 011.5, A15.0, Z86.11 |
| Rheumatoid arthritis | 714.0, 714.2, 714.81, M06.9, M06.00, M06.011-M06.012, M06.019, M06.021-M06.022, M06.029, M06.031-M06.032, M06.041-M06.042, M06.049, M06.051-M06.052, M06.059, M06.061-M06.062, M06.069, M06.071-M06.072, M06.079, M06.08-M06.09 |
| Sarcoidosis | 135, D86.0-D86.3 |
| Solid organ lung transplant | V42.0, V42.1, V42.6, V42.7, Z94.0, Z94.1, Z94.2, Z94.3, Z94.4 |
| Sjögren’s syndrome | 710.2, M35, M35.00-M35.09 |

**Supplementary Figure 1: NTM-PD incidence trends by region using 1 claim incidence definition.**


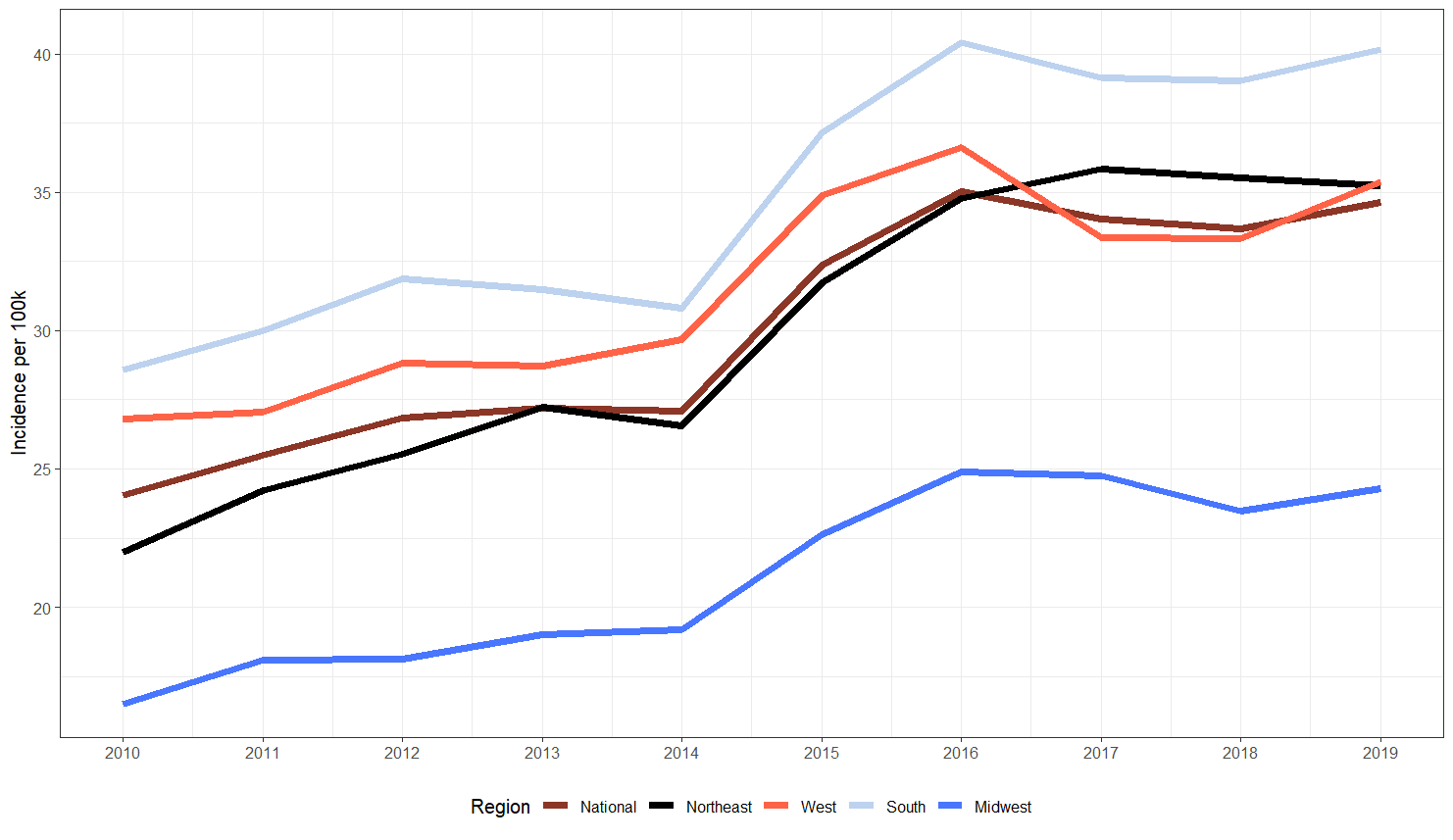

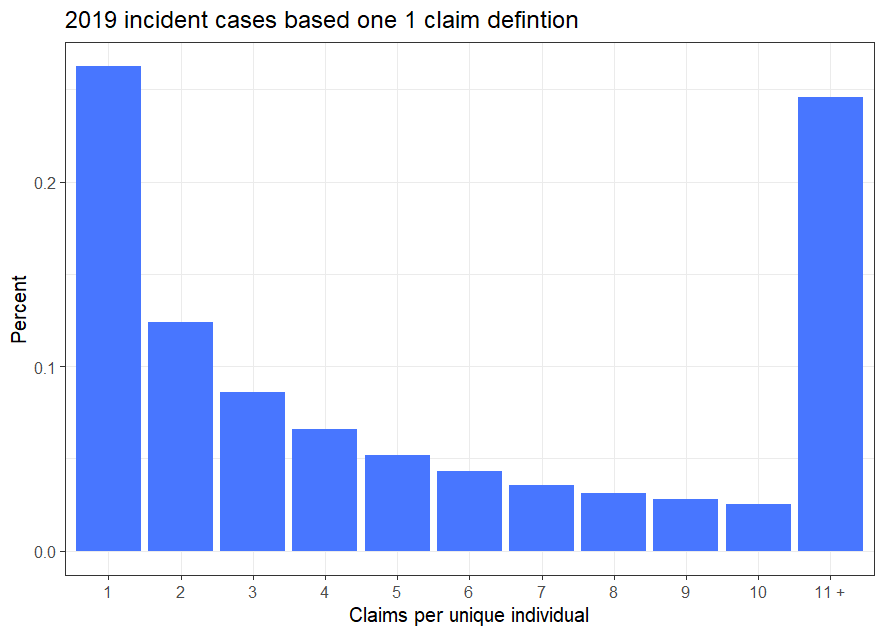


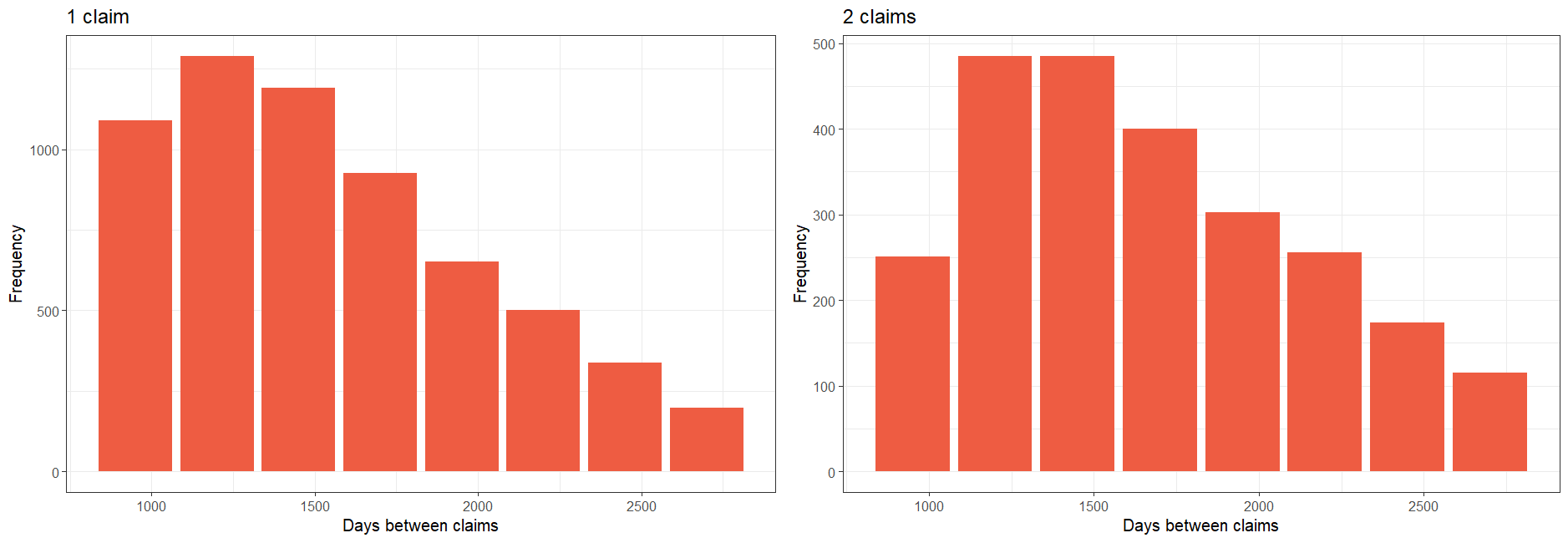


We identified 89,587 incident cases from 2010-2019 using the 1 claim definition. National incidence rose from 24.0 per 100k persons in 2010 to 34.6 in 2019 (top left). In 2019, individuals meeting the case definition had on average 8.3 claims per person and 73.8% of individuals has at least 2 claims (top right). We then compare time between claims using 1 vs 2 claim definition (bottom). We found that the time between claims was 308.6 using the 1-claim definition and 773 using the 2-claim definition.

**Supplementary Figure 2: Age-specific average yearly incidence by sex from 2010-2019.**


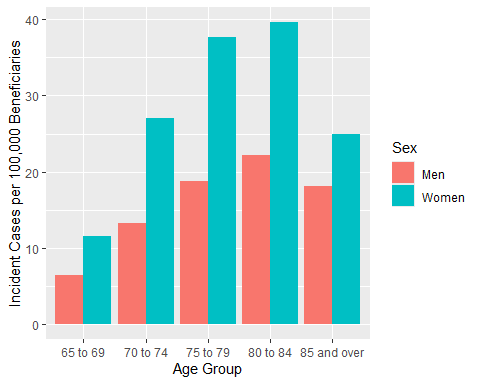


Incidence per 100,000 persons for men and women by 5-year age groups over the study period, 2010-2019.
